# Facteurs prédictifs de l’échec de traitement antituberculeux en Guinée Conakry

**DOI:** 10.11604/pamj.2015.22.146.7216

**Published:** 2015-10-15

**Authors:** Souleymane Nimagan, Regis Gothard Bopaka, Mamadou Mouctar Diallo, Boubacar Djelo Diallo, Mamadou Bailo Diallo, Oumou Younoussa Sow

**Affiliations:** 1 Service de Pneumo-Phtisiologie de Ignace Deen, Guinée Conakry; 2 Service de Pneumologie CHU de Brazzaville, Brazzaville, Congo

**Keywords:** Facteurs prédictifs, Echec, Antituberculeux, Tuberculose, predictive factors, failure, antituberculosis, Tuberculosis

## Abstract

La tuberculose est un véritable problème de santé publique. C'est une maladie guérissable et cette guérison passe par une bonne prise en charge thérapeutique. Il arrive parfois on assiste à l’échec thérapeutique, d'où l'intérêt de notre étude portant sur les facteurs prédictifs de ses échecs. Dans l'espace d'une année sur 1300 cas de tuberculose toute forme confondue, 700 cas de tuberculose pulmonaire à microscopie positive ont été répertorié dont 100 cas transférés. La tranche d’âge de 15-25 ans a été la plus touchée avec un sexe-ratio de 2 en faveur des hommes et 41,66% de nos malades ont été les ouvriers suivis de 20,83% des commerçants. La majorité de nos patients provenait de Conakry soit 99, 5%. Sur 600 patients suivis les nouveaux cas représentaient 83,33% et l’échec thérapeutique représentait 12 cas soit 2%. L'interruption du traitement représente le principal facteur de l’échec. Les facteurs qui ont influencé la régularité des malades au traitement ont été multiples. Des facteurs liés à l'organisation du système de santé, la rupture des médicaments antituberculeux, l’éducation sanitaire insuffisante, les contraintes de la supervision du traitement, l'implication insuffisante et la vente des médicaments par le personnel de santé. Des facteurs liés aux patients eux-mêmes, la crainte de perte d'emploi, les contraintes financières. Les renforcements de l'organisation du système sanitaire et l’éducation thérapeutiques pourront réduire le taux d’échec du traitement antituberculeux. L'amélioration de la qualité de la prise en charge des malades en situation d’échec devrait passer par une culture systématique des expectorations avec antibiogramme.

## Introduction

La tuberculose est une maladie contagieuse causée par le mycobacterium tuberculosis. Elle se propage par voie aérienne. Il s'agit d'un véritable problème de santé publique. Selon les estimations de l'Organisation Mondiale de la Santé (OMS), près de 9,4 millions de nouveaux cas de tuberculose ont été déclarés et près de 1,7 millions de patients sont décédés [1]. Cependant, des problèmes thérapeutiques sont rencontrés et parmi eux l’échec du traitement antituberculeux [2–4]. Le taux d’échec de traitement anti tuberculeux en Guinée est passé de 1% en 2004 à 2% en 2011 [4]. En Guinée il y'a peu de données relatives aux facteurs prédictifs de l’échec de traitement antituberculeux. Cette croissance progressive avec le risque de survenue d'une tuberculose multi résistante, est une des raisons qui nous a motivé le choix de notre travail.

## Méthodes

Il s'agit d'une étude prospective de type descriptif d'une durée d'un an allant du 1er janvier au 31 décembre 2012, après avoir obtenu l'accord de comité d’éthique. Tous les patients suivis (externes et hospitalisés) dans le service pour la tuberculose ont été enregistré. Les patients admis au service de pneumo-phtisiologie pour la tuberculose pulmonaire étaient inclus dans l’étude. La tuberculose pulmonaire à microscope négative avec culture négative et les formes extra pulmonaires étaient exclus de l’étude.

Les variables d’études étaient le sexe, l’âge, la profession (fonctionnaire, ouvriers/artisanat, ménagère, cultivateur, commerçant, élève/étudiant, chauffeur et sans emploi), la provenance urbaine ou rurale, les résultats de l'examen direct et de culture des expectorations à la recherche de bacille de Koch. Les variables thérapeutiques étaient regroupées en catégorie I et II (Tableau 1) [5, 6]. La catégorie I est le cas le traitement pour la première fois. Il s'agit du nouveau cas. Elle est destinée aux malades tuberculeux qui n'ont jamais été traités auparavant (ou ayant été traités moins d'un mois). Le traitement dure 6 mois. La catégorie II est le cas du retraitement, destinée aux malades tuberculeux ayant été traités auparavant. Elle dure pendant 8 mois [6].

**Tableau 1:** Indication des régimes de traitements

Catégorie de traitement Cas de tuberculose	Régimes thérapeutiques	
**1. Nouveau cas**	Phase intensive	Phase de continuation
Tuberculose pulmonaire à frottis positif	2 RHZE	4 RH
Tuberculose pulmonaire à frottis négatif		
Tuberculose extra pulmonaire		
**2. Tuberculose pulmonaire à frottis positif**	2 SRHZE	5RHE
Rechute	1RHZE	
Reprise après abandon		
Echec		
**3. Tuberculose chronique ou multirésistante**	Association des *médicaments* mineurs réservés aux centres de références	

**R:** rifampycine; **H:** isoniazide; **Z:** pyrazinamide; **E:** éthambutol; **S:** streptomycine

Il existe des termes qui nécessite la précision comme : la tuberculose pulmonaire à microscopie positive (TPM+) est un malade qui a au moins deux échantillons de microscopie positifs ou un échantillon positif des expectorations et une radiographie pulmonaire avec des images évocatrices de tuberculose pulmonaire ou un échantillon positif et une culture positive; la tuberculose pulmonaire à microscopie négative (TPM-) est un malade qui présente au moins deux échantillons d'expectorations sans bacille de Koch ayant des anomalies radiologiques évocatrices de tuberculose avec absence de réponse au traitement antibiotique à large spectre, pour lesquels une chimiothérapie antituberculeuse a été prescrite; la tuberculose extra pulmonaire (TEP) qui est une autre localisation de la tuberculose (tuberculose des séreuses : plèvres, méninges, péritoines etc…); la rechute de tuberculose est un cas de malade déclaré guéri ou traitement terminé de toute forme de tuberculose par

un médecin dans le passé, après une chimiothérapie complète, et qui présente de nouveau des frottis positifs dans les expectorations; la reprise après abandon est le cas de tout patient interrompant son traitement pendant 2 mois ou plus et qui retourne dans un service de santé avec des frottis positifs dans les expectorations ; la guérison est un cas d'un patient chez qui deux examens bactériologiques successifs aux 5 ^ème^ et 6 ^ème^ mois de traitement sont négatifs (nouveau cas); 5^ème^ et 8 ^ème^ mois pour les cas en retraitement ; le traitement terminé est un cas de tout malade qui a régulièrement terminé son traitement mais sans avoir fait tous les examens bactériologiques qui prouvent sa guérison; l’échec du traitement est un malade ayant des frottis positifs cinq mois ou plus après le début du traitement ou ayant un frottis positif après 2 mois de chimiothérapie pour TPM(-); perdus de vue est un malade qui a abandonné son traitement pendant deux mois ou plus ; transféré est un malade qui été transféré dans une autre unité d'enregistrement pour lequel le résultat du traitement est inconnu ; décès est un cas d'un malade décédé quelle que soit la cause au cours du traitement antituberculeux. Les données étaient recueillis à partir des fiches d'enquêtes pré établies après le consentement éclairé des malades de manière confidentielle et anonymes, puis analysées à l'aide du logiciel Epi infos 3.3.2, saisis à l'aide du logiciel Word 2010. Le seuil de significativité retenu était de 5%.

## Résultats

**Figure 1:**
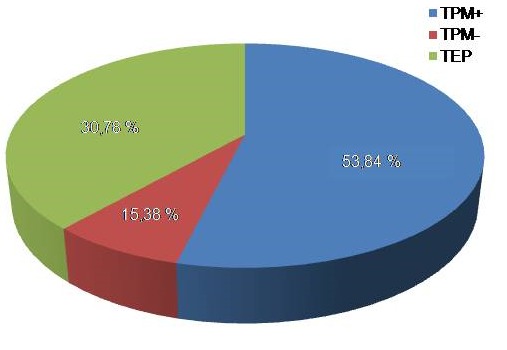
Fréquence de la tuberculose

Au cours de la période d’étude allant du 1 janvier au 31 décembre 2012, les 1300 cas de tuberculose toutes formes confondues, 900 (69,22%) de forme pulmonaire dont 700 cas de TPM+ ont été colligés (Figure 1). Parmi les 700 patients pris en charge pour TPM+, 100 patients ont été transférés. Sur les 600 patients restants, les hommes représentaient 66,7% contre 33,3% des femmes avec un sex- ratio de 2. La moyenne d’âge de nos patients a été de 34,13 ± 24 ans avec des extrêmes de 15 à 88 ans. La tranche d’âge la plus touchée a été de 15 à 25 ans (Tableau 2). Les ouvriers représentaient 41,66%, suivi de 20,83% des commerçants (Tableau 3). L'origine urbaine a été de 99,5 % en provenance de Conakry contre 0,5 % hors Conakry. Sur 600 patients suivis les nouveaux cas représentaient 83,33% et l’échec thérapeutique a été noté dans 2% soit 12 cas (Tableau 4). Les 12 cas ont été tous les ouvriers de sexe masculin. Les nouveaux cas ont été 10 et 2 cas pour le retraitement. La culture réalisée chez 8 patients en échec à la recherche des résistances aux antituberculeux a été négative. L'interruption a été le facteur principal de l’échec de traitement dans 10 cas soit 83,33% pour des raisons suivantes, la vente des médicaments anti tuberculeux, le déplacement fréquent de ces patients, des horaires incompatibles et les contraintes professionnelles. Dans 2 cas 16,67% les facteurs de l’échec ont été la crainte de perte d'emploi, les contraintes financières de ces patients.

**Tableau 2:** Répartition des patients selon l’âge

Tranche d’âge	Effectifs	Pourcentage
15-25	300	42,85+
26-35	190	27,14
36-45	100	14,28
46-55	70	10
> 55	40	5,71
**Total**	**600**	**100**

+ Différence significative avec p< 0,005

**Tableau 3:** Répartition selon les catégories socio professionnelles

Classes	Effectifs	Pourcentage
Ouvriers	250	41,66
Commerçant/ Marchant	125	20,83
Elève	50	8,33
Chauffeur	30	5
Ménagère	35	5,83
Fonctionnaire	35	5,83
Cultivateur	20	3,33
Etudiant	30	5
Sans emploi	25	4,16
**Total**	**600**	**100**

**Tableau 4:** Répartition des patients selon les types de TPM+

Types de TPM +	Effectif	Pourcentage
Nouveau cas	500	83,33%
Rechute	50	8,33%
Reprise après abandon	38	6,34%
Echec	12	2%
Total	600	100%

## Discussion

La tuberculose demeure un véritable problème de santé publique surtout dans les pays en voie de développement [6–9]. La prise en charge est multifactorielle. Elle est élaborée dans chaque pays en fonction des programmes de lutte contre cette maladie. En Guinée d'après ce programme en 2004 on notait 2% d’échec thérapeutique comme dans notre série. Nous avons éprouvés des difficultés dans la collecte des données et l'obtention des consentements des patients. Ils faillaient plus du temps à expliqués aux patients l'importance de ce travail. Ce travail à des limites car il s'agit d'une série hospitalière. Néanmoins ce travail est original du fait qu'il met en exergue non seulement les défaillances des comportements des patients mais également les défaillances du personnel, et du système de santé. Cela n'enlève en rien les efforts que fournissent les systèmes sanitaires et les organisations non gouvernementales dans cette lutte contre la tuberculose. Le Programme Nationale de Lutte Contre la Tuberculose instaure la gratuitement du traitement.

Au cours de la période d’étude, nous avons colligé 1300 cas de tuberculose toutes formes confondues dont 69,22% de forme pulmonaire. Parmi les 700 patients pris en charge pour TPM+, 500 nouveaux cas soit 71,42% et soit 83,33% sur 600 patients non transférés. Cette prédominance de nouveau cas pourrait s'expliquer par le manque de l'information et de l'ignorance de ces malades par rapport à leurs maladies malgré tous les efforts fournis par le ministère de la santé les médias et les organisations non gouvernementales ouvrant dans la lutte contre la tuberculose.

La moyenne d’âge de nos patients était de 34,13 ± 24 ans avec la tranche d’âge la plus touchée était de 15 à 25 ans soit une fréquence de 41,66%. La moyenne et la tranche d’âge de nos patients est jeune. Certains auteurs trouvent la moyenne d’âge similaire respectivement à 33±16 ans et 35 ans [4, 9]. Cependant d'autres auteurs trouvent la moyenne d’âge plus élevée à 39 ± 16,3 ans [8]. Le sexe masculin était prédominant dans notre série. Plusieurs auteurs trouvent également cette prédominance masculine [8–10]. L'origine urbaine qui représente qui presque la totalité de nos patients provenait de Conakry. Au Maroc les auteurs trouvent aussi l'origine urbaine prédominante [9]. Cette fréquence élevée pourrait s'expliquer par le fait que notre service est l'unique structure de référence pour la prise en charge des tuberculeux. Le sexe masculin et l'origine urbaine sont incriminés comme facteurs prédictifs de mauvaise observance thérapeutique [9].

Les ouvriers ont été la couche socio professionnelle la plus touchée avec une fréquence de 41,66%. Les ouvriers travaillent en groupe avec un contage tuberculeux non négligeable même si ces derniers n'acceptent pas qu'ils sont en contacts avec les cas tuberculeux. D'où l'intérêt de compagne d'information des populations cibles car la perception populaire de cette maladie dans le comportement et le recours au soin de santé est considérable [11]. Certains auteurs ne trouvent pas la différentielle professionnelle dans la mauvaise prise en charge [8, 9].

Les 83,33% des cas d'interruptions étaient observés chez les patients mis sous un régime de six mois. Cette fréquence élevée dans notre série pourrait s'expliquer par le manque d'information par rapport à la durée du traitement et sur le danger de l'abandon, la vente des médicaments antituberculeux, la rupture des stocks de médicament antituberculeux. Les patients ont interrompu le traitement dans la crainte de perte d'emploi et les contraintes financières. L'arrêt du traitement est un facteur prédictif d’échec du traitement et d'autres auteurs constatent qu'il s'agit aussi d'un facteur prédictif de récidive de la tuberculose pulmonaire [10]. D'après ces patients si on signale à nos employeurs ils risquent de nous licenciés et les absences pour les prises médicamenteuses pourront avoir des répercussions sur le salaire. Tous ceux-ci témoignent les failles des programmes comme soulignent aussi d'autres auteurs [9]. En Côte d'Ivoire les auteurs sonnaient déjà l'alarme en disant la stratégie de prise en charge des cas d’échec au régime de catégorie 1 doit être révisée dans le souci de prévenir l’éclosion de la tuberculose multi résistante [7]. Le développement continu et la généralisation de l'Approche Pratique de la Santé Respiratoire ou Practical Approach to lung Health ont été notifiés par certains auteurs [12–14] afin d'améliorer la prise en charge diagnostique et thérapeutique.

## Conclusion

L'interruption représentait le principal facteur d’échec de traitement, cependant, les raisons principales étaient, la vente des médicaments antituberculeux, la rupture des médicaments anti tuberculeux, les contraintes professionnelles et financières. La perte de l'emploie des chez les ouvriers qui ne déclarent pas leur maladie chez ces employés est à souligné. L'amélioration de la prise en charge des patients en situation d’échec doit passer nécessairement par la réalisation systématique des cultures et des expectorations [15].
